# Editorial: Cognitive-motor development and its consequences in children with neurodevelopmental disorders

**DOI:** 10.3389/fcogn.2024.1417227

**Published:** 2024-05-08

**Authors:** Yao-Chuen Li

**Affiliations:** Department of Physical Therapy, China Medical University, Taichung, Taiwan

**Keywords:** neurodevelopmental disorder, motor development, cognition, child health, comorbidity

Recent studies have indicated that the prevalence of neurodevelopmental disorders (NDDs) may either stabilize or show a slight increase over time (Yang et al., 2022; Cainelli and Bisiacchi, 2023; Li et al., 2023). It is estimated that NDDs, such as autism spectrum disorder (ASD), attention deficit/hyperactivity disorder (ADHD), specific learning disorder, or communication disorder, may affect appropriately 20% of children, although this percentage may vary across cultures, ages, or sexes (Francés et al., 2022; Yang et al., 2022; Li et al., 2023). Given this globally higher prevalence, significant attention has been directed toward understanding the underlying mechanisms and their consequences in childhood populations as these symptoms could persist from early childhood into adulthood and have lifelong adverse effects on child health (Antolini and Colizzi, 2023).

In contrast to many other childhood diseases or disorders, there is a high rate of comorbidity among NDDs with a child potentially meeting diagnostic criteria for more than two disorders (Morris-Rosendahl and Crocq, 2020). For example, up to 90% of children with ASD may concurrently have at least one other NDD, whereas developmental coordination disorder (DCD) often co-occurs with ADHD in school-aged children (Francés et al., 2022; Antolini and Colizzi, 2023). This has led researchers to consider the importance of the intertwined relationship between motor and cognitive abilities during child development. From the perspectives of child development and neuroscience, it is evident that the acquisition of motor abilities could facilitate cognitive development, with both tasks activating very similar brain areas, such as the prefrontal cortex or cerebellum (Diamond, 2000; Leisman et al., 2016; Libertus and Hauf, 2017; Veldman et al., 2019). Conversely, motor difficulties may co-occur with cognitive challenges in children with NDDs.

The co-existence of motor and cognitive problems in children with NDDs may further impact their physical and mental health, leading to issues, such as physical inactivity, poor physical fitness, or internalizing/externalizing problems. To emphasize the significance of this issue, this Research Topic aimed to enhance understanding of the relationship between cognitive and motor development and how motor-cognitive development may affect physical and mental health in preschool or school-aged children with NDDs. Additionally, the focus was on how motor-cognitive interventions may improve children's health.

Although no intervention studies were collected in this Research Topic, our collection identified that children with NDDs may encounter difficulties in performing tasks across multiple developmental domains. Through a scoping review conducted by Karimi and Nelson, children with Down syndrome aged between 0 and 18 were reported to experience developmental delays in motor and language functioning (Karimi and Nelson). Additionally, among the few studies which have investigated the cross-sectional or longitudinal motor-language link, most found a positive relationship. Similar findings were observed in children with ASD aged between 1 and 5 years, indicating that, in addition to poor communication, social, and cognitive skills, delays in gross and fine motor skills were notable, compared to typically developing children (Nordin et al.).

Interestingly, when the focusing on the adverse effects of both motor and cognitive difficulties, some children with NDDs may not be inferior to their peers. Subara-Zukic et al. investigated the performance on locomotor-cognitive dual task in children with and without DCD using the augmented reality technology and found similar results in both groups. They argued that this could result from task difficulty or the selection of gait variables, and thus recommended that further research should take these issues into account. Furthermore, some unexpected findings were noted in typically developing children, indicating that mental health may be less affected by motor difficulties in preschool children (Hirata et al.). Hirata et al. found that motor difficulties at 3–4 years failed to predict mental illness or poor prosocial skills at 5–6 years. However, as this longitudinal study only enrolled a small sample size during early childhood, further research is warranted to track this relationship over a longer period by recruiting more participants

In conclusion, motor and cognitive or language problems may co-occur in children with NDDs, such as ASD or intellectual disability. Addressing these issues requires early intervention targeting improvements in both skills and preventing their adverse health consequences. Moreover, as findings are still inconsistent in some populations, such as DCD, more research is urgently needed to provide theoretical and practical implications to better understand the underlying mechanism and guide evidence-based interventions.

## Author contributions

Y-CL: Writing – original draft, Writing – review & editing.
